# Case Report: Bow Hunter's Syndrome Caused by Compression of Extracranially Originated Posterior Inferior Cerebellar Artery

**DOI:** 10.3389/fneur.2021.756838

**Published:** 2021-10-26

**Authors:** Noriya Enomoto, Kenji Yagi, Shunji Matsubara, Masaaki Uno

**Affiliations:** Department of Neurosurgery, Kawasaki Medical School, Kurashiki, Japan

**Keywords:** posterior inferior cerebellar artery, Bow hunter's syndrome, persistent first intersegmental artery, posterior decompression, surgery

## Abstract

Bow hunter's syndrome (BHS) is most commonly caused by compression of the vertebral artery (VA). It has not been known to occur due to an extracranially originated posterior inferior cerebellar artery (PICA), the first case of which we present herein. A 71-year-old man presented with reproducible dizziness on leftward head rotation, indicative of BHS. On radiographic examination, the bilateral VAs merged into the basilar artery, and the left VA was predominant. The right PICA originated extracranially from the right VA at the atlas–axis level and ran vertically into the spinal canal. During the head rotation that induced dizziness, the right PICA was occluded, and a VA stenosis was revealed. Occlusion of the PICA was considered to be the primary cause of the dizziness. The patient underwent surgery to decompress the right PICA and VA *via* a posterior cervical approach. Following surgery, the patient's dizziness disappeared, and the stenotic change at the right VA and PICA improved. The PICA could be a causative artery for BHS when it originates extracranially at the atlas–axis level, and posterior decompression is an effective way to treat it.

## Introduction

Bow hunter's syndrome (BHS) is a transient and symptomatic vertebrobasilar insufficiency that occurs during head rotation, resulting in dizziness and fainting (1). Compression of the dominant vertebral artery (VA) at the axis–atlas level of the cervical spine is commonly considered to be the cause when reduction of blood flow through the compressed VA is not compensated as expected by blood flow from the contralateral VA (2). Compression of the non-dominant VA may also cause BHS, but this situation is rare (3). BHS is not known to be caused by compression by any other artery except the VA.

The posterior inferior cerebellar artery (PICA) is the largest branch of the VA, and its position is highly variable. In some variations, it originates extracranially at the atlas–axis level, where it is associated with an embryonic remnant of the first intersegmental artery (FIA) (4). It enters the cervical dural sac in parallel with the C2 nerve root (5). This extracranial PICA could be compressed by head rotation and thereby cause BHS, but this situation has never been reported.

We herein present a rare case of BHS that was caused by compression of an extracranially originated PICA. To our knowledge, this is the first report of BHS in which compression was caused by another artery than the VA.

## Case Report

**Figure 1:**
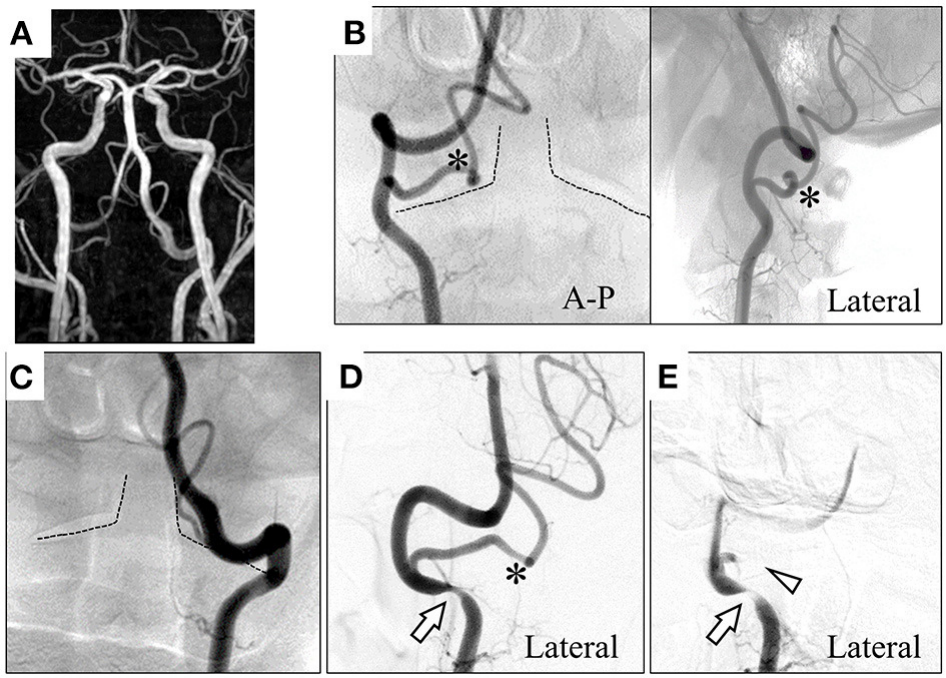
Magnetic resonance angiography (MRA) shows the bilateral vertebral arteries (VAs) merging into the basilar artery, and the left VA is dominant **(A)**. Digital subtraction angiography (DSA) shows the right posterior inferior cerebellar artery (PICA) originating and coursing into the spinal canal between the atlas and the axis **(B)**. The left VA proceeds into the spinal canal at the atlas–axis level **(C)**. On dynamic DSA at 45° leftward head rotation **(D)**, the right VA was compressed at the V3 segment. At 60° leftward head rotation **(E)**, the right PICA becomes occluded in addition to severe compression of the right VA. Dotted line: trace of axis; *: right PICA; arrow: compressed right VA; arrowhead: occluded right PICA. A–P, anterior–posterior.

**Figure 2:**
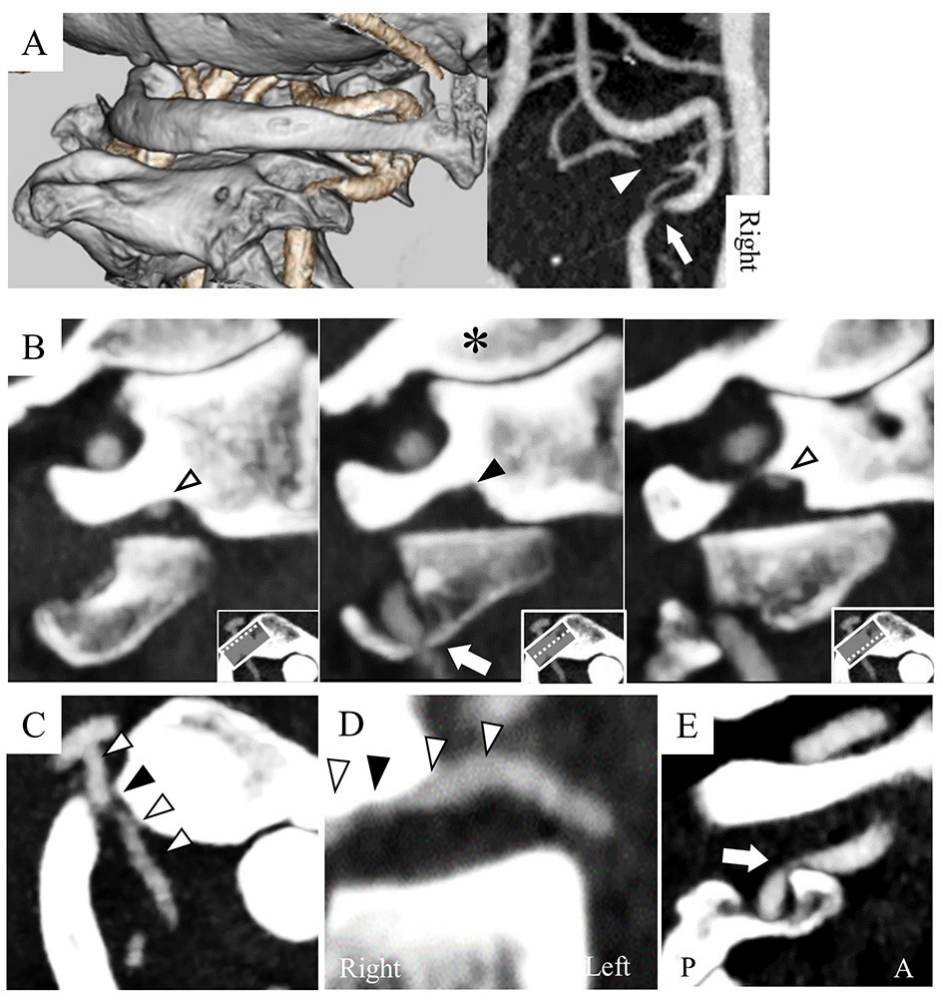
Computed tomography angiography (CTA) with leftward head rotation shows the right PICA and VA as compressed **(A)**. The PICA was compressed in the narrow space surrounded by the posterior arch and lateral mass of the atlas and the superior articular facet of the axis (**B**: sagittal image of the atlas; **C**: axial; **D**: coronal). The right VA was compressed by the anterior wall of the transverse foramen at the axis (**E**: sagittal image of the axis). White arrowhead: PICA, black arrowhead: compressed PICA, arrow: compressed VA, *: occipital condyle, A: anterior, P: posterior.

A 71-year-old man presented with a several-month history of reproducible transient dizziness on leftward head rotation, with no neurological deficit, thus indicative of BHS. Magnetic resonance angiography (MRA) showed no pathological abnormality; he exhibited bilateral VAs merging into the basilar artery, and the left VA was dominant (Figure 1A). However, digital subtraction angiography (DSA) showed variants of the bilateral vertebral arteries; the right PICA originated from the right VA at the atlas–axis level and ran vertically into the spinal canal (Figure 1B), whereas the left VA ran into the spinal canal at the atlas–axis level without passing through the atlas' foramen transversarium (Figure 1C). Leftward head rotation at 45° did not induce dizziness, but dynamic DSA showed stenosis of the right VA at the level of the axis (Figure 1D). At 60° head rotation, which induced dizziness, occlusion of the right PICA was revealed (Figure 1E). Computed tomography angiography (CTA) on leftward head rotation showed that the PICA was severely compressed in the narrow space surrounded by the atlas and axis, and that the right VA was compressed at the axis level (Figure 2). Notably, the occipital articular facet did not shift during the head rotation, which was compensated by atlantoaxial hyper-rotation in the form of atlantoaxial rotary subluxation. Thereafter, the extracranial PICA, together with the C2 nerve root and perivascular venous plexus, ran through a narrow tunnel surrounded by bone components of atlas and axis during head rotation. The compression of the PICA was mainly considered responsible for causing the BHS in the patient. However, the concomitant VA stenosis may have resulted in poor blood supply into the PICA.

**Figure 3:**
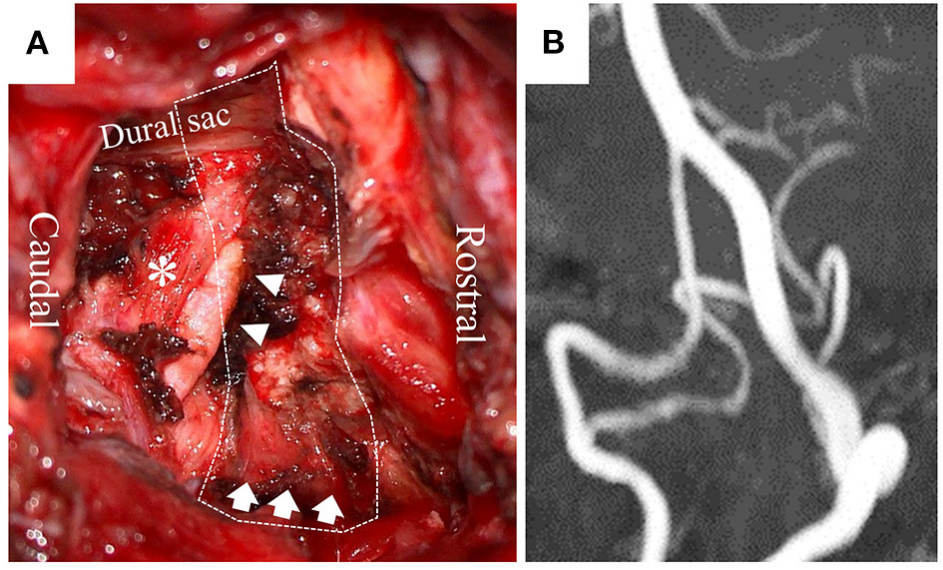
Operative view **(A)** shows a dissected right PICA (arrowhead), C2 nerve root (*), and decompressed right VA (arrow) after resecting part of the atlas (dotted line: resected atlas). Postoperative MRA at 60° leftward head rotation **(B)** shows no compression of the right PICA and VA.

The dizziness was restrictive to the patient's daily activities; therefore, he underwent surgery to decompress the right PICA and VA *via* a posterior approach (Figure 3A). A 6-cm linear skin incision was made on the back of the neck, with 3 cm laterally to the right at the level of the axis–atlas. The posterior arch of the atlas was identified following dissection between the splenius capitis and semispinalis capitis muscles. Posterior cervical muscles were preserved, including the ones originating from the atlas and axis. The right VA was identified by Doppler on the cranial side of the atlas's arch. The posterior wall of the atlas transverse foramen was removed, and the VA was dissected from the canal of transverse foramen and surrounding connective tissues, enabling the VA to shift posteriorly. Bleeding from the venous plexus was controlled by bipolar coagulation with an oxidized cellulose-based hemostatic sheet. Following resection of the right posterior arch from the transverse foramen to the spinal canal, the PICA in the C2 root sheath was identified by Doppler. By opening the sheath, the intra-sheath PICA was identified running in parallel with the C2 nerve root, and it was dissected to be totally free between the VA and dural sac. There was no fibrous band compressing the PICA.

The postoperative course was uneventful. The patient had no dizziness during head rotation, thereby freeing him from the dizziness-associated life restriction. On postoperative MRA at 60° leftward head rotation, compression of the right VA and PICA had disappeared (Figure 3B). BHS did not recur without any thrombotic therapy for 3 months after the surgery.

## Discussion

BHS has been known to be caused only by obstruction of the VA. This is the first case of BHS caused by compression of an extracranially originated PICA that was alleviated by vascular decompression.

An embryonic remnant of the FIA can be associated with VA variants at the atlas–axis level, and these are classified into three types: persistent FIA, VA fenestration, and atlas–axis originating PICA (as in the present case) (4). The frequency of atlas–axis originating PICA has been reported in up to 1.1% of the population; however, its association with BHS has not been previously reported (6, 7). Only two cases of BHS or bow hunter's stroke due to compression or injury of embryonic remnant FIA have been reported in the literature, and they affected the other two VA variations. One patient's BHS was attributed to compression of persistent FIA, and the other patient, with bow hunter's stroke, was caused by dissection of the fenestrated VA (8, 9) (Table 1).

**Table 1:** PFIA-associated Bow hunter's syndrome/stroke.

	**Case**	**Age**	**Sex**	**Type of disease**	**Pathology**	**Disposition of PFIA in vertebrobasilar system**	**Treatment**	**Outcome**
1	Yamaguchi et al. (8)	45	M	Bow hunter's stroke	Embolic stroke due to dissected PFIA	VA fenestration	Atlas-axis fusion	Good
2	Buch et al. (9)	38	M	Bow hunter's syndrome	Transient poor blood flow due to PFIA stenosis	VA running into spinal canal at atlas-axis	Decompression of artery	Good
3	Our case	71	M	Bow hunter's syndrome	Transient poor blood flow due to PFIA occlusion	Extracranially originated PICA	Decompression of artery	Good

*PFIA, persistent first intersegmental artery; VA, verterbral artery; PICA, posterior inferior cerebellar artery*.

In BHS, stenotic change of the VA is usually attributed to its compression by the transverse foramen, an osteophyte, a herniated disc, or a fibrous band (1). However, mechanisms by which an FIA-associated remnant artery can have stenotic change at head rotation are unknown. The FIA-associated remnant artery runs together with the C2 nerve root between the atlas and the axis between the posterior arch of the axis and the vertebral arch of the atlas (5). In our patient with the head rotation, the FIA-associated PICA went through an atlantoaxial rotary subluxation-forming narrow tunnel surrounded by bones (the posterior arch, the lateral mass of the atlas, and the superior articular facet of the axis) together with the C2 nerve root and the well-developed venous plexus. Hence, the atlantoaxial rotary subluxation might be one of the causes for BHS due to stenotic change at the FIA remnant. Cervical degenerative change and/or congenital abnormalities are believed to cause BHS (10). In our patient, degenerative spial change was suspected to have caused BHS by affecting the congenital vascular anomaly, given that BHS emerged at an older age.

BHS is normally caused by compression of the dominant VA where the ipsilateral decreased blood flow is not compensated by the collateral blood flow from the contralateral non-dominant VA (1). On the contrary, it is rare that compression of non-dominant VA causes BHS because of sufficient collateral flow from the contralateral dominant VA. However, a few cases have been reported to be associated with BHS because of insufficient compensatory collateral blood flow (3). In our patient, BHS was considered to be caused by compression of extracranially originated PICA. The VA stenosis at head rotation was also detected. However, the VA stenosis was not considered to be the main cause of BHS because collateral blood flow from the contralateral dominant VA presumably compensated for the poor blood flow in the ipsilateral non-dominant VA. However, this may be indirectly associated with the BHS by decreasing perfusion pressure into the occluded PICA.

To identify the causative arterial stenosis in BHS with such a vascular anomaly, careful radiographic evaluation was needed, and head rotation to the degree inducing the symptom was necessary. In our patient, the PICA occlusion was able to be identified by the sufficient rotation of the head, leading to the effective treatment.

BHS impacts daily life by causing dizziness or syncope. In addition, it may cause life-threatening conditions such as cerebral infarction and VA dissection. Conservative management and surgical treatment have been performed. For conservative management, wearing a cervical collar, refraining from extreme neck rotations, and antiplatelet therapy are applied, but the effectiveness of these procedures is unclear and difficult to maintain. On the contrary, excellent results of surgical treatment with decompression of the VA or cervical fusion have also been reported; the success rates of treatment were 87% in VA decompression alone and 100% in fusion with/without decompressing the VA (1). Although cervical fusion may cure BHS more frequently than decompression alone, restriction of neck motion could occur postoperatively, resulting in limitations in the patient's daily activities. Therefore, decompression alone, which preserves neck motion, might be a first-line therapy (2). A standard surgical strategy for FIA remnant-associated BHS has not been established because only two cases have been reported (8, 9). One patient with persistent FIA-associated BHS was successfully treated by decompression alone *via* hemilaminectomy of the atlas; the other patient who had VA fenestration as FIA remnant and presented with dissection and stroke was treated by cervical fusion. In our patient, BHS was improved by vascular decompression alone. Atlantoaxial fusion could have led to severe motion restriction at the patient's neck, because poor movement of the atlanto-occipital joint was considered to be compensated by atlantoaxial hyper-rotation. The atlas hemilaminectomy was performed to decompress the FIA-associated PICA in concert with posterior transverse foraminotomy at the atlas. The hemilaminectomy might have directly improved the rotational compression of the FIA-associated PICA. The transverse foraminotomy indirectly improves impaired blood flow in the PICA by decompressing the VA.

## Conclusion

BHS can be caused by compression of extracranially originated PICA, which can be alleviated by surgically decompressing it. In order to identify the exact causative arterial stenosis in BHS, such as the PICA, head rotation to the degree that induces the symptom might be needed during the radiographic evaluation, in order to determine the appropriate treatment.

## Data Availability Statement

The raw data supporting the conclusions of this article will be made available by the authors, without undue reservation.

## Ethics Statement

Ethical review and approval was not required for the study on human participants in accordance with the local legislation and institutional requirements. The patients/participants provided their written informed consent to participate in this study. Written informed consent was obtained from the individual(s) for the publication of any potentially identifiable images or data included in this article.

## Author Contributions

KY contributed to the conception and design of the manuscript. NE wrote the manuscript. All authors contributed to manuscript revision, read, and approved the submitted version.

## Funding

This study was partly supported by Research Grant from the Kawasaki Medical School.

## Conflict of Interest

The authors declare that the research was conducted in the absence of any commercial or financial relationships that could be construed as a potential conflict of interest.

## Publisher's Note

All claims expressed in this article are solely those of the authors and do not necessarily represent those of their affiliated organizations, or those of the publisher, the editors and the reviewers. Any product that may be evaluated in this article, or claim that may be made by its manufacturer, is not guaranteed or endorsed by the publisher.
